# Evaluation of long-term supplementation of a direct-fed microbial and enzymatically hydrolyzed yeast cell culture product on feedlot growth performance, efficiency of dietary net energy utilization, heat stress measures, and carcass characteristics in beef steers

**DOI:** 10.1093/tas/txad016

**Published:** 2023-02-03

**Authors:** Erin R Gubbels, Warren C Rusche, Elliot Block, Tom Rehberger, Jesse S Thomson, Zachary K Smith

**Affiliations:** Department of Animal Science, South Dakota State University, Brookings, SD 57007; Department of Animal Science, South Dakota State University, Brookings, SD 57007; Arm & Hammer Animal Nutrition, Church and Dwight Company, Princeton, NJ 08540; Arm & Hammer Animal Nutrition, Church and Dwight Company, Princeton, NJ 08540; Arm & Hammer Animal Nutrition, Church and Dwight Company, Princeton, NJ 08540; Department of Animal Science, South Dakota State University, Brookings, SD 57007

**Keywords:** direct-fed microbial, heat stress, yeast cell wall

## Abstract

The objective of this research was to determine the influence of long-term supplementation (258 d) of a direct-fed microbial (DFM) and yeast cell wall (YCW) product used alone or in combination on growth performance, dietary net energy utilization, and carcass characteristics in beef steers finished under climatic conditions in the Northern Plains (NP). Single-sourced Charolais × Red Angus steers [*n* = 256; body weight = 246 ± 1.68 kg] were blocked by pen location in a 2 × 2 factorial arrangement of DFM and YCW. Steers were administered a series of diets common to the NP and administered ractopamine hydrochloride (RH; 300 mg/kg) during the last 28 d of the finishing phase. Steers were vaccinated and poured at processing and individually weighed on days 1, 14, 42, 77, 105, 133, 161, 182, 230, and 258. Temperature–humidity index (THI) was calculated during RH supplementation. For 98% of the experiment, the THI was lower than 72 and thus cattle were not under high-ambient temperature. On days 1, 2, 21, and 22 of RH supplementation, respiration rates (RR), and panting scores (PS) were determined before and after AM and PM feedings (0700 h, 1100 h, 1400 h, and 1700 h). A DFM + YCW interaction was noted for the proportion of steers categorized as PS 2.0 at 1100 h on day 21 (*P* = 0.03) and RR on day 21 at 1400 h (*P* = 0.02). Control steers had a greater proportion of PS 2.0 compared to DFM or YCW steers (*P* ≤ 0.05), while DFM + YCW steers did not differ from others (*P* ≥ 0.05); DFM + YCW steers had greater (*P* < 0.05) RR compared to DFM steers, while control and YCW steers did not differ from others (*P* ≥ 0.05). No DFM + YCW interactions or main effects (*P* ≥ 0.05) were observed for cumulative growth performance measures. However, YCW steers had 2% lower (*P* = 0.04) dry matter intakes compared to steers not fed YCW. No DFM + YCW interactions or main effects (*P* ≥ 0.05) were observed for carcass traits or liver abscess severity. However, a DFM + YCW interaction (*P* < 0.05) was noted for the distribution of USDA yield grade (YG) 1 and Prime carcasses. Control steers had a greater proportion (*P* < 0.05) of YG 1 carcasses compared to other treatments. DFM+YCW steers had a greater proportion (*P* < 0.05) of USDA Prime carcasses compared to DFM or YCW but were similar to control steers, which were also similar to DFM or YCW. Overall, the use of DFM and YCW alone or in combination had minimal effects on growth performance, carcass traits, and heat stress measures in steers finished in NP climatic conditions.

## INTRODUCTION

Antimicrobials (i.e., chlortetracycline) have been used in livestock feeds in an attempt to control bovine respiratory disease (BRD) symptoms and pathogenic bacteria that reside in the gastrointestinal tract of cattle that appear healthy (i.e., asymptomatic). However, certain antimicrobials have been suggested to cause antimicrobial-resistant pathogenic populations that could pose risks to public health (Ohta et al., 2019). Therefore, on January 1, 2017, all medically important antimicrobials to human medicine were listed in the Veterinary Feed Directive (FDA, 2022). Since that time, there has been increased research in the investigation of alternative feed additives to feed cattle during all phases in the feedlot. Microbial feed additives have been used to combat health and nutritional challenges. These feed additives include *Bacillus subtilis-*based direct-fed microbials (DFM) and have been shown to reduce harmful pathogenic bacteria in the gastrointestinal tract (Krehbiel et al., 2003; Broadway et al., 2020) and improve dry matter intake (DMI) and average daily intake (ADG) during the receiving period (Smock et al., 2020). Prebiotic and probiotic feed additives with enzymatically hydrolyzed yeast cell wall (YCW) components of *Saccharomyces cerevisiae* have also improved health status and immune responses due to enhanced ruminal pH and ruminal fiber digestion (Salinas-Chavira et al., 2015). In another study, YCW supplementation helped to reduce BRD during a 30-d preconditioning period, as well as increased ADG and feed efficiency (Silva et al., 2018). These benefits may be exacerbated by the combination of probiotics and prebiotics (Colombo et al., 2021).

It is well known that the receiving period in the feedlot is one of the most stressful events in a calf’s life and can cause concerns about animal welfare and productivity (Duff and Galyean, 2007; Arthington et al., 2008). The positive effects on the growth performance of cattle that receive probiotics and/or prebiotics are commonly observed in stressed cattle, such as newly received cattle, cattle under poor management practices, or cattle under high-ambient heat load (Duff and Galyean, 2007; Silva et al., 2018; Colombo et al., 2021). In general, those benefits have been observed during short periods of supplementation (i.e., <90 d). Therefore, subsequent events that occur throughout the remainder of the time at the feedlot can also raise concerns.

A main characteristic of cattle that are finished in the Northern Plains (NP) is that for the majority of the time, there are favorable climatic conditions (without high heat ambient load) for finishing. Since the permanence of the cattle in the feedlot generally exceeds 200 d, extreme environmental stress (i.e., cold vs. heat stress) may occur and could hinder overall feedlot performance. Research has been conducted on management strategies to help mitigate environmental stress at both extremes (Mader, 2003; Smerchek and Smith, 2020). Recent research has shown that YCW may also help to reduce the impact of environmental stress and improve DMI and ADG during increased ambient temperatures (Salinas-Chavira et al., 2015; Sanchez-Mendoza et al., 2015). However, in non-stressed animals (or adapted cattle), the magnitude of the response of probiotics and prebiotics could be minimal (Uyeno et al., 2015).

The information about the effects of prebiotics, probiotics, and their combination during long-term supplementation (adapted cattle to feedlot conditions) is scarce. Thus, this information would be valuable to determine the strategies of supplementation. For this reason, the objective of this experiment was to determine the influence that a DFM and YCW fed alone or in combination have on animal growth performance, efficiency of dietary net energy (NE) utilization, carcass characteristics, and liver abscess (severity and prevalence) in beef steers fed in confinement from weaning to harvest under NP climatic conditions.

## MATERIALS AND METHODS

### Institutional Animal Care and Use Approval

This study was conducted at the Ruminant Nutrition Center (RNC) in Brookings, SD, USA. The animal care and handling procedures used in this study were approved by the South Dakota State University Animal Care and Use Committee (Approval Number: 2009-044A).

### Climatic Variables, THI, Respiration Rate, and Panting Score Measures

Climatic variables (ambient temperature, relative humidity, and wind speed) were obtained every 30 min from a weather station (VANTAGE PRO2 PLUS, Davis Instruments, Hayward, CA) located at the RNC throughout the experimental period (October 2020 through July 2021). The temperature–humidity index (THI) was calculated using the following formula: THI = 0.81 × ambient temperature + [relative humidity × (ambient temperature − 14.40)] + 46.40 (Hahn, 1999). These measurements were taken in order to verify that animals were not under stress due to a high environmental heat load.

At the initiation (days 1 and 2) of RH supplementation and the during last high ambient temperature days near the end of RH supplementation (days 21 and 22), steers were evaluated for respiration rates (RR) and panting scores (PS) at 0700 h (before AM feeding), 1100 h (after AM feeding), 1400 h (before PM feeding), and at 1700 h (after PM feeding). The respiration rate was determined on three steers per pen by a single evaluator. RR, expressed as breaths per minute (BPM) was calculated according to the following equation: BPM = 600/s required for 10 inward and outward flank movements (Hales et al., 2014). PS were based upon the following scoring system (Hales et al., 2014): 0 = No panting; 1 = Slight panting, mouth closed, no drool, easy to see chest movement; 2 = Fast panting, drool present, no open mouth; 2.5 = As for 2, but occasional open mouth panting, tongue not extended; 3 = Open mouth and excessive drooling, neck extended, head held up; 3.5 = As for 3 but with the tongue out slightly and occasionally fully extended for short periods; 4 = Open mouth with tongue fully extended for prolonged periods with excessive drooling. Neck extended and head up; 4.5 = As for 4 but head held down. Cattle “breathe” from the flank. Drooling may cease.

A single person made the visual evaluation of the 32 pens at each evaluation time point. Notes of the number of animals in each respective panting category within the pen were taken. Since the pen was considered the experimental unit, no individual animal identification was recorded. PS are expressed as a percentage of the cattle in each pen expressing that specific panting score. For example, if 6 out of 8 steers were determined to have a PS of 0 and 2 out of 8 steers had a PS of 1, then 75% of the cattle in each pen would have a PS of 0, and 25% would have a PS of 1.

### Cattle Management and Dietary Treatments

Single-sourced, newly weaned Charolais × Red Angus steers, (*n* = 302) from Western South Dakota, were transported approximately 513 km to the RNC in October 2020. Upon arrival, steers were group housed (10 steers/pen) in 7.62 m × 7.62 m concrete surface pens and were offered long-stem grass hay and ad libitum access to water.

The morning following arrival, all steers were subjected to an individual body weight (BW) measurement used for allotment purposes, application of a unique identification ear tag, vaccination against viral respiratory diseases (Bovishield Gold 5, Zoetis) and clostridial species (Ultrabac 7/Somubac, Zoetis), and administration of pour-on moxidectin (Cydectin, Bayer) according to label directions. Due to a known health history of steers from this ranch, no metaphylaxis was administered upon arrival. The afternoon following initial processing, a subset of steers (*n* = 256; BW = 246 ± 21.8 kg; selected based upon temperament, health, and uniformity of BW) was allotted to treatment pens (*n* = 8 pens/treatment with 8 steers/pen; 32 pens total).

Steers were randomly assigned to treatments using a 2 × 2 factorial arrangement of factors DFM and YCW. The resulting treatments included: 1) Fed no DFM and no YCW (Control); 2) Fed the DFM and no YCW (DFM; Certillus CP B1801 Dry, *Bacillus subtilis* and *Lactobacillus plantarum* based DFM; Arm & Hammer Animal Nutrition; 28 g/steer·d^−1^); 3) Fed no DFM and yes YCW (YCW; Celmanax, enzymatically hydrolyzed yeast component of *Saccharomyces cerevisiae*; Arm & Hammer Animal Nutrition; 18 g/steer·d^−1^); and 4) Fed the DFM and the YCW (DFM+YCW; Certillus 28 g/steer·d^−1^ and Celmanax 18 g/steer·d^−1^ of Celmanax).

The morning following processing, the first four replicate pens (pens 1–16) were weighed and treatment diets were initiated. The following morning, the last four replicate pens (pens 17–32) for each treatment were weighed and treatment diets were initiated. Two weigh days were used in order to ensure cattle in the last four replicate pens would not be confounded by time off feed due to the processing time. Each subsequent weigh day and processing event was handled in a similar manner in that the first four pen replicates for each treatment were worked the day prior to the last four pen replicates.

The initial on-test BW was the average of the two BW measures collected at processing and the day-test diets were initiated and reduced by 4% to account for digestive tract fill. Throughout the remainder of the study, individual BWs were recorded on days 14, 42, 77, 105, 133, 161, 182, 230, and 258. In order to monitor growth performance, steers were weighed at 28 ± 7 d intervals. All steers were fed in a small pipe and cable pens with concrete bunks (95.25 linear cm of bunk space/steer) and pen floors (7.25 m^2^/steer); all pens are equipped with automatic heated waters.

### Feeding Management

All diets contained monensin sodium (Rumensin-90, Elanco Animal Health) at 27.6 mg/kg during the receiving and growing phase and 33.1 mg/kg during the finishing phase; all diets were fortified with vitamins and minerals to meet or exceed nutrient requirements for growing and finishing beef steers (NASEM, 2016).

Supplements for dietary treatment inclusion were manufactured every 30 d at the SDSU feed mill in Brookings, SD. Ingredients were analyzed weekly for dry matter (DM) content and composited monthly for nutrient determination. Actual diet formulation was based upon weekly DM determination and feed batching records along with energy content (Preston, 2016) which is presented in Table 1. All steers were subjected to a 42-d receiving period. On study day 42, steers were weighed, and implanted with a Synovex-S (Zoetis) implant containing 200 mg progesterone and 20 mg estradiol benzoate (EB). Steers were fed the growing diet until day 112 where they were transitioned using two intermediate diet steps to the finishing diet that was fed until harvest (Table 1). The terminal implant (200 mg trenbolone acetate and 28 mg EB; Synovex Plus, Zoetis) was administered 97 d prior to harvest (day 161). Implant retention checks (days 77 and 182) were conducted by a trained technician. One steer from DFM + YCW was identified as having a missing implant; at this time the appropriate implant was re-administered, and no other severe implant site anomalies were noted on day 77 or day 182. Ractopamine HCl (RH) was fed at a rate of 300 mg/steer·d^−1^ for the final 28 d on feed (day 230 of study). Throughout the entire study, steers were fed twice daily (0800 h and 1400 h) in a 50:50 split. Bunks were managed according to a slick bunk management system to allow for *ad libitum* access to feed. Feed was manufactured in a stationary mixer (2.35 m^3^; four pens fed per batch), all ingredients were added into the mixer to the nearest 0.45 kg, and feed was delivered to each pen separately (weighed out of the stationary mixer to the nearest 0.45 kg) into a feed delivery wagon.

**Table 1.** Diet formulation of diets offered to steers during each period of the study^*a*^

Item	Day 1–42 (receiving)	Day 43–112 (growing)	Day 113–119 (transition 1)	Day 120–126 (transition 2)	Day 127–230 (finisher 1)	Day 231–245 (finisher 2)	Day 246–258 (finisher 3)
DRC, %	–	14.68	16.85	24.33	32.74	35.49	69.10
HMC, %	–	–	15.65	22.56	32.43	34.98	–
DDGS, %	19.21	19.70	17.19	15.13	15.03	15.29	15.11
Corn silage, %	52.15	51.65	42.61	30.34	12.25	–	–
Oat hay, %	18.96	4.75	–	–	–	–	1.99
Sorghum silage, %	–	–	–	–	–	6.80	6.46
Pelleted supplement^*b*^, %	5.82	6.54	–	–	–	–	–
Liquid supplement^*c*^, %	–	–	5.14	5.10	5.24	5.27	5.24
Treatment supplement^*d*^, %	3.87	2.70	2.57	2.55	2.32	2.12	2.10
**Diet composition**							
DM, %	47.81	50.91	49.25	56.02	68.24	71.52	77.90
CP, %	12.66	13.26	13.72	13.31	13.21	13.14	13.12
NDF, %	43.31	37.94	29.86	25.07	18.48	17.12	18.09
ADF, %	26.81	19.59	14.11	11.63	11.17	9.28	9.75
Ash, %	6.56	6.19	6.26	5.75	5.35	5.36	5.44
EE, %	2.61	2.48	3.55	3.49	3.28	3.40	3.42
NE_M_, Mcal/kg	1.73	1.86	1.93	2.01	2.11	2.12	2.07
NE_G_, Mcal/kg	1.08	1.21	1.29	1.36	1.44	1.45	1.40

^*a*^Each treatment was offered the same diet with exception to the treatment supplement.

^*b*^Pelleted supplement contained exclusively soybean hulls.

^*c*^Liquid supplement (all values except for DM on a DM basis): 69.04% DM, 41.86% crude protein, 38.38% nonprotein nitrogen, 0.95 Mcal/kg of NE for maintenance, 0.66 Mcal/kg of NE for gain, 10.89% calcium, 0.32% phosphorus, 7.00% potassium, 0.22% magnesium, 6.03% NaCl, 0.33% sulfur, 4.23 ppm cobalt, 199.88 ppm inorganic copper, 11.99 ppm iodine, 15.07 mg/kg EDDI, 83.16 ppm iron, 304.81 ppm manganese, 2.90 ppm selenium, 664.59 ppm inorganic zinc, 44,064.55 IU/kg vitamin A, 376.52 IU/kg vitamin E, and 638.6 g/mg monensin sodium.

^*d*^Soybean hull carrier supplement with either 0 g/steer (CON), Certillus at 28 g/steer·d^–1^ (DFM), Celmanax at 18 g/steer·d^−1^ (YCW), or Certillus at 28 g/steer·d^−1^ and Celmanax at 18 g/steer·d^−1^ (DFM + YCW).

### Growth Performance and Dietary Energy Calculations

Growth performance (BW, ADG, gain to feed ratio (G:F), DMI) was determined from receiving through finishing. Cumulative growth performance was based upon initial BW (average BW from initial processing and day 1 with a 4% shrink applied to account for digestive tract fill) and final BW (final BW measurement at day 258 and shrunk 4%). ADG was calculated as the difference between the final shrunk BW and the initial shrunk BW, divided by days on feed. The feed efficiency was calculated from ADG/DMI.

Applied energetics measures (observed dietary NE, the ratio of observed-to-expected dietary NE, DMI, and ADG and maintenance coefficient (MQ)) were assessed from days 1 to 258 for the cumulative post-weaning feeding period. Observed dietary NE was calculated from daily energy gain (EG; Mcal/d): EG = ADG^1.097^ × 0.0557W^0.75^, where *W* is the mean equivalent shrunk BW calculated as: [average of initial shrunk BW and ending period shrunk BW × (478/final BW at 28% empty body fatness (AFBW)), kg (NASEM, 2016)], using shrunk (4%) growth performance. Maintenance energy required (EM; Mcal/d) was calculated by the following equation: EM = 0.077BW^0.75^ (Lofgreen and Garrett, 1968) where BW is the mean shrunk BW (average of initial shrunk BW and ending period BW). Using the estimates required for maintenance and gain, the observed dietary NE_M_ and NE_G_ values (Owens and Hicks, 2019) of the diet were generated using the quadratic formula: $x=\;(-b\pm \sqrt{b^{2}-4ac})/2c$, where *x* is the NE_M_, Mcal/kg, *a* = −0.41EM, *b* = 0.877EM + 0.41DMI + EG, *c* = −0.877DMI, and NE_G_ was determined from: 0.877NE_M_ – 0.41 (Zinn and Shen, 1998; Zinn et al., 2008). The ratio of observed-to-expected NE ratio was determined from observed dietary NE for maintenance or gain/tabular NE for maintenance or gain. Expected DMI was determined based on observed ADG and equivalent BW according to the following equation (NASEM, 2016): expected DMI, kg/d = (EM/tNE_M_) + (EG/tNE_G_), where tNE_M_ and tNE_G_ are NE values based upon the tabular composition of the diet. Expected ADG (kg/d) was determined from feed available for maintenance (FFM), feed available for gain (FFG), retained energy (RE; Mcal/d), median feeding weight and equivalent BW (EqBW), where FFM = EM/tNE_M_, FFG = DMI − FFM, and RE = FFG × tNE_G_ according to the following equation: expected ADG, kg/d = (13.91 × RE^0.9116^ × EqBW^−0.6837^). MQ was determined using the following equation: MQ, Mcal/W^0.75^ = [(DMI−(EG/NE_G_))NE_M_]/W^0.75^. Cumulative tabular dietary NE values were calculated from the average of the tabular dietary NE values throughout the study.

### Carcass Trait Determination

Steers were harvested after 258 d on feed post-weaning; steers were shipped the afternoon following final BW determination and harvested the next day at a commercial harvest abattoir. Steers were commingled at the time of shipping and remained this way until harvest time. Liver abscess prevalence and severity were determined following evisceration according to the Elanco Scoring System (Elanco Animal Health; Greenfield, IN) as Normal (no abscesses), A− (1 or 2 small abscesses or abscess scars), A (2– 4 well-organized abscesses less than 1 in diameter), or A+ (1 or more large active abscesses greater than 1 in diameter with inflammation of surrounding tissue). Hot carcass weight (HCW) was captured immediately following the harvest procedure. Video image data were obtained from the packing plant for rib eye area; 12th rib fat thickness; kidney, pelvic, and heat fat (KPH); and USDA marbling scores. Yield grade (YG) was calculated according to the USDA regression equation (USDA, 1997). Dressing percentage was calculated as HCW/(final BW × 0.96). Estimated empty body fat (EBF) percentage and final BW at 28% EBF (AFBW) were calculated from observed carcass traits (Guiroy et al., 2002), and proportion of closely trimmed boneless retail cuts from carcass round, loin, rib, and chuck (Retail Yield, RY (Murphey et al., 1960)).

### Management of Pulls and Removals

All steers that were pulled from their home pen for health evaluation were then monitored in individual hospital pens prior to being returned to their home pens. When a steer was moved to a hospital pen the appropriate amount of feed from the home pen was removed and transferred to the hospital pen. If the steer in the hospital returned to their home pen, this feed remained credited to the home pen. If the steer did not return to their home pen, all feed that was delivered to the hospital pen was deducted from the feed intake record for that particular pen back to the date the steer was hospitalized. Two steers were removed from the Control group, two steers were removed from the DFM group, three steers were removed from the YCW group, and five steers were removed from the DFM + YCW group. No DFM + YCW interaction was noted for any health outcomes (*P* ≥ 0.14) in the present study.

### Statistical Analysis

Growth performance, carcass traits, and efficiency of dietary NE utilization were analyzed as a randomized complete block design using the GLIMMIX procedure of SAS 9.4 (SAS Inst. Inc., Cary, NC) with a pen as the experimental unit. The model included the fixed effect of DFM, YCW, and their interaction; the block (location) was included as a random variable. Least squares means were generated using the LSMEANS statement of SAS and treatment effects were analyzed using the pairwise comparisons PDIFF and LINES option of SAS 9.4. Distribution of USDA Yield and Quality grade data as well as liver abscess prevalence and severity were analyzed as binomial proportions in the GLIMMIX procedure of SAS 9.4 with fixed and random effects in the model as described previously. An α of 0.05 or less determined significance and tendencies are discussed between 0.05 and 0.10.

## RESULTS

### Climatic Variables

Ambient weather conditions during the course of the study are presented in Table 2. The average THI during the course of the 258-d study was 43.50. In the present study, only 216.2 mm of precipitation occurred from October 21, 2020, to July 6, 2021. Total precipitation during the course of this experiment was appreciably less than historical records. For 98% of the experiment, the THI was lower than 72. However, two separate heat events occurred during the course of the experiment. Heat event 1 occurred between days 225 and 252 (mean period THI = 69.87) of the present study in which the average THI was greater than 72 for 8-d of the 28-d period. Heat event 2 occurred between 253 and 258 (mean period THI = 72.70) in which the average THI was greater than 72 for 4-d of the 6-d period. As there were only 12 d in which the average THI was greater than 72, it appears cattle were not subjected to a high-ambient heat load during the course of the experiment.

**Table 2.** Ambient temperature (*T*_*a*_), mean relative humidity (RH), and temperature–humidity index (THI) throughout the course of the experiment.

Period^*a*^	Mean *T*_*a*_, (°C)	Mean RH, (%)	Mean THI^*b*^	Days with THI >72	Wind speed, KPH	Total precipitation, mm
1	1.46	73.79	37.38	0	15.6	45.7
2	−1.06	75.91	33.71	0	13.0	0.0
3	−5.06	81.53	26.40	0	13.6	7.6
4	−8.17	79.59	22.16	0	16.5	10.9
5	−6.53	73.78	26.07	0	13.1	6.1
6	4.51	66.75	42.79	0	17.1	50.3
7	6.62	66.00	46.37	0	16.3	37.1
8	13.95	65.71	57.57	0	14.1	40.1
9	23.30	53.53	69.87	8	12.4	18.3
10	24.90	58.92	72.70	4	9.4	0.0
Average^*c*^	5.39	69.55	43.50	12	14.1	216.2

^*a*^ Each period represents 28 d, except for period 10 that represent 6 d.

^*b*^ THI = 0.81 × ambient temperature + [relative humidity × (ambient temperature − 14.40)] + 46.40.

^*c*^Average of the 258-d study, except for days with THI >72 and precipitation, which is total days with THI >72 and total precipitation during the course of the 258-d study.

### Respiration Rate and Panting Score

**Figure 1.**
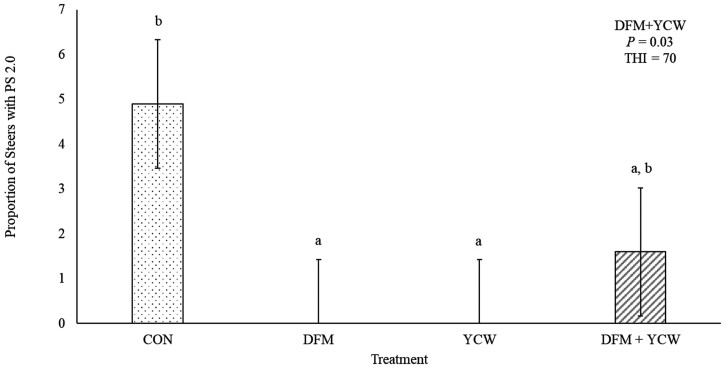
The proportion of steers with PS 2.0 at 1100 h day 21 of RH supplementation for steers fed no direct-fed microbial (DFM) or yeast cell wall (YCW) product (Control); fed the DFM and fed no YCW (DFM; Certillus CP B1801 Dry; *Bacillus subtilis, Lactobacillus plantarum;* 28 g/steer·d^−1^); fed no DFM and fed YCW (YCW; Celmanax; 18 g/steer·d^−1^); fed the DFM and the YCW (DFM+YCW; Certillus 28 g/steer·d^−1^ and Celmanax 18 g/steer·d^−1^ of Celmanax). ^a, b^ means differ (*P* ≤ 0.05).

**Figure 2.**
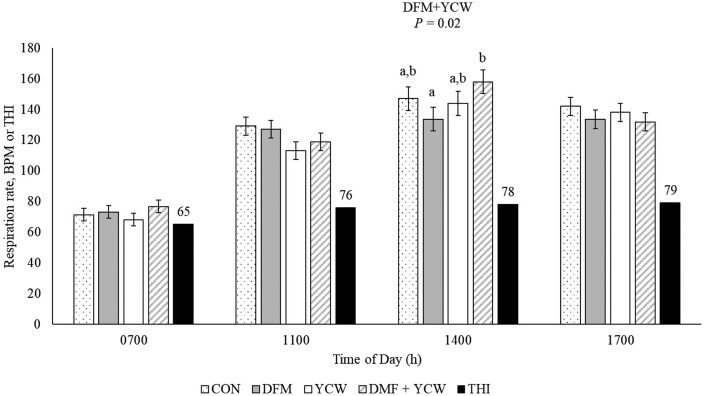
Respiratory rates on day 21 of RH supplementation for steers fed no direct-fed microbial (DFM) or yeast cell wall (YCW) product (Control); fed the DFM and fed no YCW (DFM; Certillus CP B1801 Dry; *Bacillus subtilis, Lactobacillus plantarum;* 28 g/steer·d^−1^); fed no DFM and fed YCW (YCW; Celmanax; 18 g/steer·d^−1^); fed the DFM and the YCW (DFM+YCW; Certillus 28 g/steer·d^−1^ and Celmanax 18 g/steer·d^−1^ of Celmanax). No DFM + YCW interactions (*P >* 0.05) were observed at 0700, 1100, or 1700 h. ^a, b^ means differ (*P* ≤ 0.05).

A DFM + YCW interaction was noted for the proportion of steers categorized as PS 2.0 at 1100 h on day 21 (*P* = 0.03; Figure 1) and respiration rate on day 21 at 1400 h (*P* = 0.02; Figure 2). No other interactions between DFM and YCW were noted for any other heat stress measurement (*P* ≥ 0.08). Steers from Control had more steers classified at PS 2.0 at 1100 h on day 21 compared to steers from DFM or YCW (*P* ≤ 0.05), while steers from DFM + YCW were intermediate, not differing from others (*P* ≥ 0.10). On day 21 steers from DFM + YCW had greater (*P* < 0.05) respiration rate compared to steers from DFM, steers from Control and YCW were intermediate, not differing from others (*P* ≥ 0.10).

Steers from DFM tended to have an increased respiration rate by 7.5% (*P* = 0.08) compared to steers not fed DFM at 0700 h on day 21. However, steers from DFM tended (*P* = 0.10) to have a reduced respiration rate by 8.7% compared steers not supplemented with DFM at 1700 h on day 21. Supplementation of DFM had no other appreciable influence on any measures of heat stress in the present study (*P* ≥ 0.12). Steers from YCW had a 78.3% reduction in the proportion of steers classified as PS 2.0 at 1100 h on day 1 (*P* = 0.04). Steers from YCW had a 9.4% reduction in respiration rate compared to steers not supplemented with YCW at 1100 h on day 21 (*P* = 0.01). Supplementation with YCW tended (*P* ≤ 0.10) to increase PS 1.0 at 1700 h by 8.8% and reduce PS 2.0 by 61.5% at 1700 h on day 21. Steers from YCW tended (*P* = 0.08) to have a reduction in the proportion of steers classified as PS 1.0 by 13.6% at 1400 h on day 22. The use of YCW had no other influence on any heat stress measures in the present experiment (*P* ≥ 0.12).

### Growth Performance and Dietary Net Energy Measures

Cumulative growth performance responses are presented in Table 3. No DFM + YCW interaction was noted (*P* ≥ 0.27) for live weight, ADG, DMI, G:F, observed dietary NE, the ratio of observed-to-expected dietary NE, DMI, and ADG or MQ. The use of DFM had no influence (*P* ≥ 0.15) on live weight, ADG, DMI, G:F, observed dietary NE, the ratio of observed-to-expected dietary NE, DMI, and ADG or MQ.

**Table 3.** Cumulative growth performance responses through the 258-d feeding period for the effect of DFM, YCW, and their interaction^*a*^

	Treatments^*b*^					*P* value		
Item	Control	DFM	YCW	DFM+YCW	SEM	YCW	DFM	Interaction
Pens, *n*	8	8	8	8	–	–	–	–
Steers, *n*	62	62	61	59	–	–	–	–
Initial body weight (BW), kg	246	246	247	246	–	–	–	–
Final BW, kg	656	655	649	656	7.57	0.55	0.56	0.46
Average daily gain (ADG), kg	1.59	1.59	1.56	1.59	0.029	0.52	0.48	0.43
Dry matter intake (DMI)^*c*^, kg	9.41	9.41	9.21	9.28	0.108	0.04	0.62	0.64
G:F (ADG/DMI)	0.169	0.169	0.169	0.171	0.0021	0.26	0.54	0.47
Dietary net energy for maintenance (NE_M_), Mcal/kg	1.96	1.96	1.98	1.99	0.017	0.07	0.60	0.66
Dietary net energy for gain (NE_G_), Mcal/kg	1.31	1.31	1.33	1.34	0.015	0.07	0.61	0.66
Observed-to-expected measures^*d*^								
Dietary NE_M_	1.00	1.00	1.01	1.01	0.008	0.07	0.61	0.66
Dietary NE_G_	1.00	1.00	1.01	1.02	0.011	0.07	0.60	0.66
DMI	1.00	1.00	0.99	0.99	0.010	0.08	0.54	0.66
ADG	1.00	1.00	1.01	1.03	0.016	0.08	0.48	0.62
Maintenance coefficient^*e*^, Mcal/BW^0.75^, kg	0.078	0.077	0.075	0.074	0.002	0.06	0.59	0.66

^*a*^A 4% pencil shrink was applied to all BW measures to account for gastrointestinal tract fill.

^*b*^Treatments: Fed no direct-fed microbial (DFM) or yeast cell wall (YCW) product (Control); Fed the DFM and fed no YCW (DFM; Certillus CP B1801 Dry; *Bacillus subtilis, Lactobacillus plantarum;* 28 g/steer·d^−1^); Fed no DFM and fed YCW (YCW; Celmanax; 18 g/steer·d^−1^); Fed the DFM and the YCW (DFM + YCW; Certillus 28 g/steer·d^−1^ and Celmanax 18 g/steer·d^−1^ of Celmanax).

^*c*^YCW main effect. Steers offered YCW had decreased DMI compared to steers not offered YCW (9.25 kg vs. 9.41 kg) (*P* < 0.04).

^*d*^Observed-to-expected measures: 1.00 = steers performed as expected, <1.00 = steers performed worse than expected and > 1.00 = steers performed better than expected.

^*e*^Maintenance coefficient: 0.077 = steers performed as expected, >0.077 = steers were less metabolically efficient and <0.077 = steers were more metabolically efficient.

Steers from YCW had reduced (*P* ≤ 0.04) intake by 1.8%. Observed dietary NEm and NEg tended (*P* ≤ 0.07) to be increased by 1.2% to 1.5% for YCW compared to non-supplemented controls. The ratio of observed-to-expected dietary NEm and NEg tended (*P* ≤ 0.07) to be increased by 1.0% to 1.5% for YCW compared to non-supplemented steers. The ratio of observed-to-expected DMI tended (*P* = 0.08) to be decreased by 1.0% for YCW compared to non-supplemented steers. The ratio of observed-to-expected ADG tended (*P* = 0.08) to be decreased by 2.0% for YCW compared to non-supplemented steers. The MQ was reduced by 3.9% when YCW was supplemented compared to non-supplemented controls.

### Carcass Traits

Carcass trait responses are presented in Table 4. A DFM + YCW interaction (*P* = 0.02) was noted for the distribution of USDA yield grade (YG) 1 carcass. Steers from the control had a greater (*P* ≤ 0.05) proportion of carcasses classified as YG1 compared to all other treatments. Additionally, a DFM + YCW interaction (*P* = 0.04) was noted for the distribution of USDA Prime carcasses. Steers from DFM + YCW had a greater (*P* ≤ 0.05) proportion of carcass classified as USDA Prime compared to DFM and YCW, while steers from Control were intermediate, not differing (*P* ≥ 0.10) from others. No other DFM + YCW interactions were noted (*P* ≥ 0.06). Supplemental DFM had no appreciable impact on any carcass trait responses (*P* ≥ 0.15). The use of YCW resulted in a 31.9% reduction in carcasses classified as USDA Average Choice (*P* = 0.05). Supplementation of YCW during the entire post-weaning production phase had no other effect on any carcass trait measures collected in the present experiment.

**Table 4.** Carcass trait responses for the effect of DFM, YCW, and their interaction^*a*^

						*P* value		
Item	Control	DFM	YCW	DFM+YCW	SEM	YCW	DFM	Interaction
Hot carcass weight (HCW), kg	427	425	421	425	5.03	0.49	0.75	0.43
DP^*b*^, %	64.88	64.83	64.95	64.82	0.216	0.84	0.58	0.79
Ribeye area, sq cm	99.03	98.84	98.83	99.10	0.175	0.95	0.97	0.81
Twelveth rib fat thickness, cm	1.19	1.24	1.27	1.27	0.064	0.34	0.68	0.78
Marbling^*c*^	492	484	481	481	18.1	0.59	0.74	0.74
Kidney pelvic heart fat, %	1.75	1.79	1.78	1.77	0.026	0.76	0.40	0.27
Calculated YG^*c*^	2.70	2.73	2.72	2.75	0.075	0.69	0.57	0.96
RY^*d*^, %	51.17	51.09	51.11	51.06	0.162	0.71	0.54	0.89
EBF^*e*^, %	29.86	29.91	29.94	30.03	0.376	0.71	0.78	0.95
AFBW^*f*^, kg	636	633	628	632	8.1	0.37	0.93	0.50
**Yield grade distribution, %**								
1	12.9^*a*^	1.6^*b*^	3.3^*b*^	4.9^*b*^	2.55	0.23	0.07	0.02
2	48.7	73.4	62.5	57.3	7.53	0.88	0.21	0.06
3	38.4	23.4	34.2	36.0	6.75	0.54	0.34	0.23
4	0.0	1.6	0.0	1.7	1.18	0.93	0.17	0.93
**Quality grade distribution, %**								
Select	19.6	13.0	14.5	18.8	4.96	0.94	0.81	0.25
Low choice	38.4	44.8	52.2	46.9	6.63	0.21	0.93	0.35
Average choice	27.5	35.9	23.0	20.2	5.62	0.05	0.56	0.26
High choice	11.2	6.3	10.3	10.1	3.96	0.69	0.49	0.52
Prime	3.3^*a, b*^	0.0^*b*^	0.0^*b*^	3.9^*a*^	1.68	0.88	0.88	0.04

^*a*^Treatments included: Fed no direct fed microbial (DFM) or yeast cell wall (YCW) product (Control); Fed the DFM and fed no YCW (DFM; Certillus CP B1801 Dry; *Bacillus subtilis, Lactobacillus plantarum;* 28 g/steer·d^−1^); Fed no DFM and fed YCW (YCW; Celmanax; 18 g/steer·d^−1^); Fed the DFM and the YCW (DFM + YCW; Certillus 28 g/steer·d^−1^ and Celmanax 18 g/steer·d^−1^ of Celmanax).

^*b*^Dressing percent = (HCW/final BW shrunk 4%) × 100.

^*c*^400 = small^00^

^*d*^USDA yield grade according to the regression equation described by USDA (1997).

^*e*^Retail yield as a percentage of HCW according to Murphey et al. (1960).

^*f*^Empty body fat and adjusted final body weight calculated according the equations described by Guiroy et al. (2002).

^*a, b*^indicate significance among treatments (*P* ≤ 0.05).

## DISCUSSION

### Climatic Variables, Respiration Rate, and Panting Score

The THI can be used as a measurement to analyze the relationship between climate variables and heat stress measures (Lockard et al., 2020). It has been shown to be a good indicator of heat stress severity (Lockard et al., 2020). THI levels greater than 74 have been associated with causing heat stress in cattle (Salinas-Chavira et al., 2015), and values between 70 and 74 for an extended period of time indicate that cattle could experience heat stress (Lockard et al., 2020). The present study evaluated levels greater than 72, due to a wide range of temperatures experienced throughout the duration of the study (−32.78 °C to 36.08 °C). There were only 12 d where the average THI was greater than 72 for the duration of the study, which coincided with the RH supplementation period. This is consistent with the other data in the Northern Plains where limited days were observed with high THI values (Smith et al., 2021).

Limited heat stress effects were observed in the present study. In a 76 d study in southeast Texas, steers were exposed to THI levels above 70 for 29 consecutive days (Lockard et al., 2020). During this time, decreased energy intake, ADG, and feed efficiency were observed. Steers that were offered a yeast-based additive complex had improved feed efficiencies, which seems to follow the same trend in the present study. Sanchez-Mendoza et al. (2015) also discussed under certain condition yeast supplementation may help to reduce fluctuations in daily feed intake. Perhaps yeast-based additives may help mitigate the effects of heat stress by not reducing performance. However, the THI values observed during the experiment proved that cattle were finished under favorable climatic conditions. This is further confirmed by the measures of respiration rate and PS, which overall classified their values as being non-heat stressed conditions.

### Growth Performance and Dietary Net Energy Measures

This study did not note any appreciable differences in growth performance measures. Similar to these findings, Salinas-Chavira et al. (2015) also observed no effects on growth performance during a 139 d period in the desert Southwest, while Lockard et al. (2020) observed no effects on growth performance during a 76 d period in southeast Texas. Pancini et al. (2020) also noted little to no differences when Angus steers were supplemented with a yeast culture product with monensin in Virginia for 150 d or 160 d. Although no differences in the present study were observed during the receiving phase (6.2 kg for all treatments; *P* ≥ 0.60) when crossbred steers in New Mexico were offered an enzymatically hydrolyzed yeast product and *Bacillus subtilis* product, increased DMI was observed during the first 3 wk of the receiving period (Colombo et al., 2021).

In contrast to the present study, reports of increased DMI and ADG have been observed in crossbred steers in the desert Southwest that were offered an enzymatically hydrolyzed yeast product (Salinas-Chavira et al., 2015) plus a chromium-enriched yeast product (Sanchez-Mendoza et al., 2015), as well as in calf-fed Holstein steers in the desert Southwest (Salinas-Chavira et al., 2018). The increased ADG was attributed to increased DMI. Although decreased DMI was observed for YCW steers in the present study, there were no differences in ADG or feed efficiency. Perhaps this provides a possible explanation for the tendency of improved observed-to-expected values for steers offered the YCW supplement. It seems improvements in ADG can be attributed to differences in increased energy intake, rather than improved energy efficiency (Sanchez-Mendoza et al., 2015). It is important to note that Salinas-Chavira et al. (2015) observed increased DMI when the average THI was 80, and Salinas-Chavira et al. (2018) observed increased ADG when the average THI was 75.2. Since cattle in the present study were not under high ambient heat stress during this time, this may be an indicator that cattle supplemented with yeast products may respond better under conditions of stress. This could be in part due to yeast supplementation making ruminal fermentation more stable through the reduction in the variation of ruminal ammonia concentrations and increased concentrations of cellulolytic bacteria (Harrison et al., 1988). This reduction in the fluctuation of ruminal fermentation could be an agent in promoting overall ruminal health.

### Carcass traits

No appreciable differences were observed in the present study, which is consistent with studies evaluating enzymatically hydrolyzed yeast cell effects on carcass traits (Salinas-Chavira et al., 2015; [Bibr CIT0011][Bibr CIT0018]). However, one study indicated that increased ADG translated to increased HCW in calf-fed Holsteins offered the enzymatically hydrolyzed yeast cell product (Salinas-Chavira et al., 2018). Another study also noted increased dressing percentage and tendencies for lower calculated YGs and a lower percentage of A+ liver scores in control black-hided steers compared to steers offered a yeast-based additive complex (Lockard et al., 2020). When crossbred steers were supplemented with a yeast product with chromium, steers had increased REA, and tendencies for decreased fat thickness, and increased retail yield (Sanchez-Mendoza et al., 2015). However, it was noted the differences were likely due to the addition of chromium with the yeast product component.

## CONCLUSION

The use of DFM and YCW had minimal effects on overall growth performance or carcass traits. Cumulative growth performance measures may be enhanced by the use of YCW. The basis for this improvement requires further study but may be attributed to cattle responding to yeast supplementation under conditions of stress, which could have influenced ruminal health through the reduction of ruminal bacterial fluctuations and may have resulted in a tendency for a reduced MQ requirement. However, the DFM and YCW used alone or in combination in relatively unstressed animals cannot be expected to show productive performance benefits. Further investigation is warranted to determine the overall effects on performance and heat stress measures of cattle offered enzymatically hydrolyzed YCW components.

## Data Availability

Data can be made available with a reasonable request to Z.K.S.

## DISCOLSURES

Authors E.R.G., W.C.R., and .Z.K.S. declare no conflict of interest other than the fact that Arm & Hammer Animal Nutrition provided partial funding for this research. Authors E.B., T.R., and J.S.T. are employed by Arm & Hammer Animal Nutrition.

## References

[CIT0001] Arthington, J. D., X. Qiu, R. F. Cooke, J. M. B. Vendramini, D. B. Araujo, C. C. Chase, and S. W. Coleman. 2008. Effects of preshipping management on measures of stress and performance of beef steers during feedlot receiving. J. Anim. Sci. 86:2016–2023. doi:10.2527/jas.2008-0968 18407994

[CIT0002] Broadway, P. R., J. A. Carroll, N. C. B. Sanchez, T. R. Callaway, S. D. Lawhon, E. V. Gart, L. K. Bryan, D. J. Nisbet, H. D. Hughes, J. F. Legako, et al. 2020. *Bacillus subtilis* PB6 supplementation in weaned holstein steers during an experimental salmonella challenge. Foodborne Pathog. Dis. 17:521–528. doi:10.1089/fpd.2019.2757 32349549

[CIT0003] Colombo, E. A., R. F. Cooke, A. P. Brandao, J. B. Wiegand, K. M. Schubach, C. A. Sowers, G. C. Duff, E. Block, and V. N. Gouvea. 2021. Performance, health, and physiological responses of newly received feedlot cattle supplemented with pre- and probiotic ingredients. Animal. 15. doi:10.1016/j.animal.2021.100214 34029789

[CIT0004] Duff, G. C., and M. L. Galyean. 2007. Board-invited review: recent advances in management of highly stressed, newly received feedlot cattle. J. Anim. Sci. 85:823–840. doi:10.2527/jas.2006-501 17085724 PMC7109667

[CIT0005] FDA. 2022. Veterinary feed directive (VFD). United States Department of Agriculture Food and Drug Administration, Washington D.C.

[CIT0006] Guiroy, P. J., L. O. Tedeschi, D. G. Fox, and J. P. Hutcheson. 2002. The effects of implant strategy on finished body weight of beef cattle. J. Anim. Sci. 80:1791–1800. doi:10.2527/2002.8071791x 12162646

[CIT0007] Hahn, G. L. 1999. Dynamic responses of cattle to thermal heat loads. J Anim Sci. 77:10–20. doi:10.2527/1997.77suppl_210x 15526777

[CIT0008] Hales, K. E., S. D. Shackelford, J. E. Wells, D. A. King, M. D. Hayes, T. M. Brown-Brandl, L. A. Kuehn, H. C. Freetly, and T. L. Wheeler. 2014. Effects of feeding dry-rolled corn-based diets with and without wet distillers grains with solubles and zilpaterol hydrochloride on performance, carcass characteristics, and heat stress in finishing beef steers1. J. Anim. Sci. 92:4023–4033. doi:10.2527/jas.2014-7638 25023799

[CIT0009] Harrison, G. A., R. W. Hemken, K. A. Dawson, R. J. Harmon, and K. B. Barker. 1988. Influence of addition of yeast culture supplement to diets of lactating cows on ruminal fermentation and microbial populations 1. J. Dairy Sci. 71:2967–2975. doi:10.3168/jds.S0022-0302(88)79894-X 3230186

[CIT0010] Krehbiel, C. R., S. R. Rust, G. Zhang, and S. E. Gilliland. 2003. Bacterial direct-fed microbials in ruminant diets: performance response and mode of action12. J. Anim. Sci. 81(14_suppl_2):E120–E132. doi:10.2527/2003.8114_suppl_2E120x

[CIT0011] Lockard, C. L., C. G. Lockard, D. M. Paulus-Compart, and J. S. Jennings. 2020. Effects of a yeast-based additive complex on performance, heat stress behaviors, and carcass characteristics of feedlot steers. Livest. Sci. 236. doi:10.1016/j.livsci.2020.104052

[CIT0012] Lofgreen, G. P., and W. N. Garrett. 1968. A system for expressing net energy requirements and feed values for growing and finishing beef cattle. J. Anim. Sci. 27:793–806. doi:10.2527/jas1968.273793x

[CIT0013] Mader, T. L. 2003. Environmental stress in confined beef cattle1. J. Anim. Sci. 81(14_suppl_2):E110–E119. doi: 10.2527/2003.8114_suppl_2E110x

[CIT0014] Murphey, C. E., D. K. Hallett, W. Tyler, and J. C. Pierce. 1960. Estimating yields of retail cuts from beef carcasses. In: American Society of Animal Production, Chicago, IL. p 1–12.

[CIT0015] NASEM National Academies of Sciences, Engineering, and Medicine. 2016. Nutrient requirements of beef cattle. 8th ed. The National Academies Press, Washington, DC.

[CIT0016] Ohta, N., B. Norby, G. H. Loneragan, J. Vinasco, H. C. den Bakker, S. D. Lawhon, K. N. Norman, and H. M. Scott. 2019. Quantitative dynamics of *Salmonella* and *E. coli* in feces of feedlot cattle treated with ceftiofur and chlortetracycline. PLoS One. 14:e0225697. doi:10.1371/journal.pone.0225697 31791047 PMC6887520

[CIT0017] Owens, F. N., and R. B. Hicks. 2019. Can net energy values be determined from animal performance measurements? A review of factors affecting application of the California Net Energy System. Transl. Anim. Sci. 3:929–944. doi:10.1093/tas/txy130 32704857 PMC7252571

[CIT0018] Pancini, S., R. F. Cooke, A. P. Brandao, N. W. Dias, C. L. Timlin, P. L. P. Fontes, A. F. F. Sales, J. C. Wicks, A. Murray, R. S. Marques, et al. 2020. Supplementing a yeast-derived product to feedlot cattle consuming monensin: impacts on performance, physiological responses, and carcass characteristics. Livest. Sci. 232. doi:10.1016/j.livsci.2019.103907

[CIT0019] Preston, R. L. 2016. 2016 feed composition table BEEF magazine. [accessed February 1, 2019]. https://www.beefmagazine.com/sites/beefmagazine.com/files/2016-feedcomposition-tables-beef-magazine.pdf

[CIT0020] Salinas-Chavira, J., C. Arzola, V. Gonzalez-Vizcarra, O. M. Manriquez-Nunez, M. F. Montano-Gomez, J. D. Navarrete-Reyes, C. Raymundo, and R. A. Zinn. 2015. Influence of feeding enzymatically hydrolyzed yeast cell wall on growth performance and ­digestive function of feedlot cattle during periods of elevated ambient temperature. Asian Australas. J. Anim. Sci. 28:1288–1295. doi:10.5713/ajas.15.0061 PMC4554869 26194225

[CIT0021] Salinas-Chavira, J., M. F. Montano, N. Torrentera, and R. A. Zinn. 2018. Influence of feeding enzymatically hydrolysed yeast cell wall plus yeast culture on growth performance of calf-fed Holstein steers. J. Appl. Anim. Res. 46:327–330. doi:10.1080/09712119.2017.1299742

[CIT0022] Sanchez-Mendoza, B., A. Montelongo-Terriquez, A. Plascencia, N. Torrentera, R. A. Ware, and R. A. Zinn. 2015. Influence of feeding chromium-enriched enzymatically hydrolyzed yeast on growth performance, dietary energetics and carcass characteristics in feedlot cattle under conditions of high ambient temperature. J. Appl. Anim. Res. 43:390–395. doi:10.1080/09712119.2014.978781

[CIT0023] Silva, L. G. T., R. F. Cooke, K. M. Schubach, A. P. Brandao, R. S. Marques, T. F. Schumaher, P. Moriel, and D. W. Bohnert. 2018. Supplementing a yeast-derived product to enhance productive and health responses of beef steers. Animal. 12:1576–1583. doi:10.1017/S1751731117003585 29277170

[CIT0024] Smerchek, D. T., and Z. K. Smith. 2020. Bedding application to feedlot steers: influence on growth performance, estimated maintenance coefficient, carcass characteristics, and circulating metabolites in beef steers. Animals (Basel). 10. doi:10.3390/ani10101766 PMC7600082 33003554

[CIT0025] Smith, Z. K., P. R. Broadway, K. R. Underwood, W. C. Rusche, J. A. Walker, N. C. Burdick Sanchez, J. A. Carroll, D. Lafleur, and J. E. Hergenreder. 2021. Evaluation of *Bacillus subtilis* PB6 on feedlot phase growth performance, efficiency of dietary net energy utilization, and fecal and subiliac lymph node *Salmonella* prevalence in spring placement yearling beef steers fed in southeastern South Dakota1,2,3. Transl. Anim. Sci. 5. doi:10.1093/tas/txab002 PMC7881255 33604519

[CIT0026] Smock, T. M., K. L. Samuelson, J. E. Hergenreder, P. W. Rounds, and J. T. Richeson. 2020. Effects of *Bacillus subtilis* PB6 and/or chromium propionate supplementation on clinical health, growth ­performance, and carcass traits of high-risk cattle during the feedlot receiving and finishing periods. Transl. Anim. Sci. 4(3):12. doi:10.1093/tas/txaa163 PMC7584392 33134873

[CIT0027] USDA. 1997. Official United States standard for grades of beef carcasses agric. Marketing, USDA Washington DC.

[CIT0028] Uyeno, Y., S. Shigemori, and T. Shimosato. 2015. Effect of probiotics/prebiotics on cattle health and productivity. Microbes Envir. 30:126–132. doi:10.1264/jsme2.ME14176. PMC4462921 26004794

[CIT0029] Zinn, R. A., A. Barreras, F. N. Owens, and A. Plascencia. 2008. Performance by feedlot steers and heifers: daily gain, mature body weight, dry matter intake, and dietary energetics. J. Anim. Sci. 86:2680–2689. doi:10.2527/jas.2007-0561 18539825

[CIT0030] Zinn, R. A., and Y. Shen. 1998. An evaluation of ruminally degradable intake protein and metabolizable amino acid requirements of feedlot calves. J. Anim. Sci. 76:1280–1289. doi:10.2527/1998.7651280x 9621934

